# The health of detainees and the role of primary care: Position paper of the European Forum for Primary Care

**DOI:** 10.1017/S1463423622000184

**Published:** 2022-05-16

**Authors:** Peter Groenewegen, Anja Dirkzwager, Anke van Dam, Dina Massalimova, Coral Sirdifield, Lauren Smith

**Affiliations:** 1 Nivel - Netherlands Institute for Health Services Research, PO box 1568, 3500 BN Utrecht, The Netherlands; 2 NSCR – Netherlands Institute for the Study of Crime and Law Enforcement, Amsterdam, The Netherlands; 3 AFEW International, Amsterdam, The Netherlands; 4 AFEW Kyrgyzstan, Bishkek, Kyrgyzstan; 5 Community and Health Research Unit, School of Health and Social Care, University of Lincoln, Lincoln, UK; 6 University of Lincoln, School of Psychology, College of Social Science, Lincoln, UK

**Keywords:** continuity of care, organisation of care, primary care, prison health

## Abstract

This position paper aims to increase awareness among primary care practitioners and policymakers about the specific and complex health needs of people who experience incarceration. We focus on the importance of primary care and of continuity of care between prison and community. We highlight what is known from the literature on the health of people who experience incarceration, on the organisation of prison health care, and on the role of primary care both during and after detention. We present three case descriptions of detainees’ encounters with the organisation of prison health care in three European countries. Finally, we describe the position that the European Forum for Primary Care takes. Prisoners and ex-prisoners have a worse physical and mental health compared with a cross-section of the population. However, access to good quality treatment and care is often worse than in the outside situation. In particular, well-organised primary care in the prison context could benefit prisoners and, indirectly, society at large. Moreover, continuity of care between the community and the prison situation needs improvement.

## Introduction

With this position paper, we aim to increase awareness among primary care practitioners and policymakers about the specific and complex health needs of people who experience incarceration.^1^ Whether they know it or not, primary care practitioners meet people who have been incarcerated in their practice and have patients in their practice who are at risk of being incarcerated in the future. Our aim is to discuss and suggest ways to improve primary care for (ex-)detainees, both during and after detention.

While imprisonment is the most severe sanction that can be imposed upon people in most democratic countries, it is not an uncommon sanction. On any given day, about 1.7 million people are detained in Europe, and globally, this number raises to more than 10 million people (Walmsley, 2018). Furthermore, it has been estimated that about six million people are detained in the European Region every year (World Health Organization, 2019). The vast majority of them will be released and return to the community.

Prison populations generally include some of the most vulnerable and disadvantaged people in society (Penal Reform International, 2015). Scientific evidence suggests that a selective group of persons – that is those coming from disadvantaged backgrounds with poor educational, financial, housing, and employment situations – is likely to be incarcerated (World Health Organization, 2014; World Health Organization, 2019). In addition, it is well documented that individuals with poor health are largely overrepresented in prison populations.

Primary care providers play a central role in people’s health and health care use both in the general population and in the prison context because they are often the first health care provider people turn to. Furthermore, primary care has been shown to reduce poor health and mortality (Starfield, 1994; Macinko *et al*., 2003; Starfield *et al*., 2005; Kringos *et al*., 2013). For instance, receiving regular and optimal primary care has been linked to earlier treatment, better (chronic) disease management, and increased receipt of preventive care. Moreover, consistent primary care contacts among both persons with and without a detention experience have been associated with decreased hospitalisations and emergency departments visits (Weber *et al*., 2005; Young *et al*., 2015; Green *et al*., 2016). Hence, access to good primary care during and after incarceration is of utmost importance.

Before making a number of recommendations on how to improve primary care for (ex-)detainees, we will provide background information to illustrate the problem. This will be done in two ways. First, we will highlight what’s known from the literature on the health of people who experience incarceration, on the organisation of prison health care, and on the role of primary care both during and after detention. Second, we will present three case studies of detainees’ encounters with the organisation of prison health care in three European countries.

### The health of (former) detainees

People who experience detention have a high burden of physical and mental health problems. Compared with the general population, (former) detainees are more likely to experience a variety of psychiatric and substance use-related disorders, chronic diseases, communicable diseases (e.g. HIV, tuberculosis, hepatitis, and sexually transmitted diseases), intellectual disabilities, and stress-related physical illnesses (e.g. hypertension, chest pain, chronic headaches) (Bingswanger *et al*., 2009; Fazel and Baillargeon, 2011; Wildeman and Muller, 2012; Massoglia and Pridemore, 2015; Dolan *et al*., 2016).

Moreover, high levels of comorbidity are observed in prison populations. For instance, comorbid mental health, substance use, and personality disorders are common among detainees (Butler *et al*., 2011; Young *et al*., 2018; Mundt and Baranyi, 2020). In addition, some research also showed a high co-occurrence between mental and substance use disorders on the one hand, and physical conditions or poor physical functioning on the other hand (Eytan *et al*., 2011; Barry *et al*., 2014; Semenza and Grosholz, 2019). The relatively poor health of detainees also extends to the ultimate detrimental health outcome, that is, premature death. Studies consistently showed that – compared with age and gender-matched general populations – detainees are at increased risk of mortality after their release (Binswanger *et al*., 2007; Dirkzwager *et al*., 2012; Bukten *et al*., 2017). These elevated mortality rates are particularly pronounced during the first weeks post-release. Common causes of mortality among former detainees appear to be drug overdose, cardiovascular disease, homicide, and suicide.

While prison populations in general experience relatively poor health, a number of specific high-risk groups have been identified. For instance, females, older detainees, juveniles and ethnic minorities face particular and complex health needs and barriers to health care, which makes them particularly vulnerable. Finally, the elevated prevalence of health problems is observed both before, during, and after detention (Wildeman & Muller, 2012). Given the fact that most people are released at some point and that a substantial part of them experience multiple detention spells, the World Health Organization has emphasised that detainees’ health and prison health care are an important part of public health (World Health Organization, 2009).

In sum, detainees have complex health needs. Many of their problems can be addressed by good quality health care, both during detention and afterwards, upon return to the community.

### The organisation of health care during incarceration

Different international human rights guidelines and standards have emphasised the right to health and health care of people placed in detention (UN Office on Drugs and Crime, 2013; World Health Organization, 2019). Incarcerated people cannot choose their own doctor or health care provider and have to rely on the authorities to ensure their health. States, therefore, have a special duty of care for detainees and are accountable for avoidable health damage caused by insufficient health care measures. A basic assumption of prison health care is that incarcerated persons are entitled to care and treatment that is at least equivalent to the care and treatment available in the community (i.e. the principle of equivalence). As mentioned in the Mandela Rules (rule 24.1), incarcerated people “should enjoy the same standards of health care that are available in the community, and should have access to necessary health-care services free of charge without discrimination on the grounds of their legal status” (UN Office on Drugs and Crime, 2015).

European Member States reaffirmed several important elements of prison health services, for instance, that these services should include: access to a doctor during detention without unnecessary delays; equivalence of care; gender-specific health care services; the importance of patient’s consent and confidentiality; preventive health care; professional independence; and professional competence (UN Office on Drugs and Crime, 2013). However, shortcomings of prison health care in Europe have been identified as well, such as a shortage of qualified health care personnel, prison health services that are sometimes inferior to public health services, medical tasks being carried out by non-medical staff, no or a delayed medical intake after arrival in detention, and failure to carry out a comprehensive drug policy (Pont and Harding, 2019; UN Office on Drugs and Crime, 2013).

Prison health care can be governed in different ways; the responsibility for health care in prisons can lie with the ministry of health, with the ministry of justice, or can be a shared responsibility of the ministry of health and another ministry (McLeod *et al*., 2020; UN Office on Drugs and Crime, 2013). The WHO recommends that health ministries should be responsible for prison health care services, and that prison health services are independent of prison administrations, are integrated into national health policies and systems, and are not involved in punishments of detainees (World health Organization, 2019). One of the advantages of having the health ministry responsible for prison health care is that this ensures full professional independence of health care staff and helps to avoid role conflict. Professional independence is crucial for a trustful doctor–patient relationship that is respectful of the principles of confidentiality and privacy. Having health ministries in charge of prison health may also facilitate the continuity of care for people transitioning between prisons and the community (UN Office on Drugs and Crime, 2013).

In most European countries, the ministry of justice is in charge of prison health, but in some countries, prison health care is partially integrated in the national health care system (World Health Organization, 2019). Using data collected as part of the Health in Prisons European Database (HIPED), McLeod and colleagues provide an overview of the ministries responsible for prison health in 39 European countries (see Table 1 in McLeod *et al*., 2020). They show the diversity in prison health systems in European countries, and also discuss countries that transferred the responsibility for prison health services from the ministry of justice to the ministry of health. At present, however, accurate evidence on the impact of such a transfer or on the relative benefits of different prison health care systems in general is limited (Pont and Harding, 2019).

A study in Spain also addressed the shift of the responsibility for prison health care from the Ministry of Justice to the Ministry of Health (Bengoa *et al*., 2018). The main outcome was that the coordination between primary care inside the prison and specialist care outside worked better in the new prison health care governance system, which indicates better coordination of care. However, the design of the study was not very strong.

Finally, a study by the Belgian Healthcare Knowledge Centre compared the governance of prison care in four European countries (i.e. France, the Netherlands, Scotland, Switzerland), and provides a description of (primary) care in prisons in these countries and identifies some areas for improvement (Dubois *et al*., 2017). The French prison health care system is governed by the Ministry of Health. The organisation of care is strongly connected to hospitals. As a consequence, there is not much attention paid to health promotion and prevention. The infrastructure of electronic medical records and information exchange is under-developed. There is a lack of continuity of care beyond the prison stay. Scotland transferred the governance of prison health care to the Ministry of Health in 2011, after a period of preparations. The focus of their system is on primary care, with a strong role for nurses and an emphasis on improving equity. Continuity of care is organised in a project called ‘Throughcare’; however, according to the report continuity of care remains a challenge (see also MacDonald *et al*., 2012). The Swiss system differs between cantons, and in the study, one canton with responsibility at the Ministry of Justice and one with responsibility at the Ministry of Health are described. This allows for a direct comparison within a common context. Strong points of the Ministry of Health system are independence of health care staff and the development of a health care policy; which can make working in prison more attractive. Finally, the Netherlands has a system that falls under the responsibility of the Ministry of Justice. Care is organised in multidisciplinary psycho-medical teams, with managers and nurses employed by the prison service and physicians contracted. Nurses ideally have an additional 1-year training in prison health care. The electronic medical record system in prisons is in need of improvement.

No conclusion is reached about the merits of either system. How a transfer to the Ministry of Health will work out depends – according to the report – on the way policy is implemented and the pre-existing context of care. However, the report summarises the main challenges of prison health care in general in the four countries as related to ensuring the quality of care, tackling health inequalities, and meeting the specific needs of prisoners. All in all, the authors conclude that there is a need for comprehensive primary care in prisons including effective collaboration between health care and social care (Dubois *et al*., 2017).

### Primary care in prisons

Health care in prisons is mostly primary care. Countries differ in the way this is organised and staffed, with large differences in the numbers of health care staff in relation to the number of prisoners. The HIPED database gives an average of 31.7 healthcare staff per 1000 incarcerated persons (2014–2016). Average ratios for the different categories of staff were: 10.3 for physicians, 5.0 for psychologists, 1.3 for psychiatrists, and 1.3 for dentists. However, there is large variation between countries in staff numbers and in their actual availability (e.g. during out of office hours). Primary care is also less available for pre-trial detainees compared to detainees in correctional facilities.

In the above-mentioned study of Dubois and colleagues (2017) on prison care in four European countries, a number of common characteristics and shortcomings in the organisation of primary care in prisons are discussed. First, primary care is often not provided by physicians specialised in family medicine/general practice. This seems related to problems with attracting physicians to work in the prison context. Apparently, prison health care is not seen as an attractive career option among physicians. As a consequence, nurses play an important role and prison health care depends, much more than primary care outside prisons, on nurses. This can be both positive and negative, depending on the education and training of nurses and on having short links with GPs and other physicians. In some countries, there is a special education for nurses who work in prisons (e.g. The Netherlands). Second, workforce shortages and lack of adequate training are mentioned for all four countries in this study, which may further reflect the unattractiveness of prison health care as a career option. Medical education may play a positive role in increasing the attractiveness of a career in prison health, for example, by providing internships in prisons (Brooker *et al*., 2018; Pont and Harding, 2019). Third, different countries experience challenges with having enough and adequate mental health care available. This is striking, particularly given the high mental health needs of prison populations. Fourth, out-of-office hours care is often unavailable; and continuity of care between primary and specialist care is often hampered by transportation and security rules for care that needs to be provided outside the prison. This restricts access to more specialised care. Fifth, a key element of primary care – providing person-centred care that respects the privacy of prisoners – is often hampered by overcrowding of prisons (Dubois *et al*., 2017) or by characteristics of the prison setting where patient involvement, participation, and self-management may conflict with the prisons regimes that value individual initiatives less (De Viggiani, 2006).

As it turns out, the literature on the organisation of care in prisons is rather general and focusses on the responsibility for care and governance in general. It is not specific enough to draw conclusions regarding the merits of different organisational principles and on whether or not prison health care matches the core principles of good primary care.

### Primary care during and after detention and continuity of care between prison and community care

While access to primary care has been identified as an important determinant of positive health outcomes in (former) prison populations (Weber *et al*., 2005; Young *et al*., 2015; Green *et al*., 2016), knowledge on primary care utilisation among people who experience incarceration is still limited.

The literature on prison health care tends to focus on specific interventions and their effectiveness and not on the key generic characteristics of strong primary care, namely access, comprehensiveness, continuity, coordination, and community orientation of care (Starfield, 1994; Kringos *et al*., 2010). In their broad systematic review, Kouyoumdijan and colleagues (2015) reviewed randomised controlled trials of interventions aimed at improving the health of people during detention and in the year post-release. They identified 95 studies that examined interventions focussing on substance use, mental health, infectious diseases, health services use, and chronic diseases (Kouyoumdjian, Mcisaac *et al*., 2015). Interventions included motivational interviewing, educational and skills-building interventions, psychotherapy, pharmacological interventions, vaccination (e.g. Hepatitis B vaccination), interventions to reduce sexual risk behaviour, case management, and chronic disease interventions. A positive health effect was observed in 59 studies (e.g. for mental health, substance use, infectious diseases, and health care utilisation). The authors identified some serious gaps in knowledge, such as a lack of evidence on interventions addressing chronic diseases, injuries, and reproductive health, and a lack of studies examining interventions targeted at incarcerated adolescents and women. Given the multi-morbidity of health problems and diseases in incarcerated populations, the authors of the review critically reflected on the focus on disease-specific outcomes and interventions in most studies, and concluded that: “Interventions to strengthen health systems, including primary health care during imprisonment and at time of release, might more effectively address the complex needs of this population.” (Kouyoumdjian, Mcisaac *et al*., 2015, pp. e17).

While a number of studies showed substantially elevated levels of primary care use in prison when compared with the general population, few examined primary health care use after release (Marshall *et al*., 2001; Feron *et al*., 2005; Carroll *et al*., 2017). Recent studies in Canada and Australia showed that, compared with the general population, former detainees showed increased rates of primary care use post-release as well (Carroll *et al*., 2017; Kouyoumdjian *et al*., 2019). However, other studies demonstrated that people who experience incarceration were less likely to use primary care both before and after detention, and that a substantial part of the people who experience incarceration did not use primary care either in prison or post-release (Kouyoumdjian *et al*., 2019). For instance, a study in Canada showed that over 65% of released women and over 75% of released men did not access primary care in the first month after release (Kouyoumdjian *et al*., 2018). Therefore, despite the elevated use of primary health care during detention, people who experience incarceration are typically an underserved group while outside prison (Condon *et al*., 2007; McLeod *et al.,* 2020).

The underutilisation of (primary) health care among former detainees may not be that surprising because incarcerated populations share many characteristics with other vulnerable groups known to have poor access to primary care. For instance, patient-reported access to primary care is typically lower among patients with lower socio-economic status, younger patients, male patients, patients with a migration background (Uiters *et al*., 2009; Schäfer *et al*., 2018; Lueckmann *et al*., 2021), homeless people (Health Quality Ontario, 2016), and people without insurance coverage (Evans *et al*., 2013). It is precisely these characteristics that are highly prevalent in incarcerated populations as well. Given this, correctional facilities provide an important opportunity to provide health care to a underserved population that is hard to reach outside the prison walls. Therefore, primary care providers both within and outside prisons may play an important role by addressing the health and social needs in prison and after release.

Some initiatives for interventions to improve access to healthcare for detainees have been taken; however, such interventions have rarely been evaluated. Only about a handful of studies – in the USA and Australia – examined interventions designed to improve primary care engagement or other health care use and to reduce emergency department utilisation among individuals who were recently released from prison (Wang *et al*., 2012; Kinner *et al.*, 2013; Kinner *et al*., 2016). These studies suggest that relatively low intensity, primary care based, or case management interventions may increase the use of primary care, mental health services, and support services in the community, and may decrease emergency department use among former detainees (Wilson and Davis, 2006; Lattimore and Visher, 2009; Wang *et al*., 2012; Kinner *et al*., 2016).

Continuity of care is always important but there are several reasons why this is even more important for people who experience incarceration (Bellass *et al*., 2021). First, as mentioned above, this group of people suffers from complex health problems, including communicable diseases, chronic diseases, and mental and substance-related health issues. Second, the transitions between the community and prisons are typically associated with specific health risks, such as alcohol or drugs withdrawal on admission or overdoses after release (Winter *et al*., 2015), abrupt interruptions in essential treatment (e.g. anti-retroviral therapy) (Hassan *et al*., 2011; Springer *et al*., 2011; Gonzalez and Connell, 2014), and increased risks of post-release mortality (Binswanger *et al*., 2007). Continuity of care is also hypothesised (but not thoroughly tested) to contribute to lower recidivism rates (MacDonald *et al*., 2012). In line with the importance of continuity of care, an early review study (Watson *et al*., 2004) recommended to develop a model of prison health care organisation ‘which looks beyond the prison environment to the communities which the prison serves.’ (p.126).

The importance of continuity of care was also highlighted by the United Nations in the Mandela Rules (rule 24.2), which emphasised that prison health care services should be organised in close connection to the general health care system and in such a way that it ensures continuity of care and treatment, including for HIV, other infectious diseases, and drug dependency (UN Office on Drugs and Crime, 2015). Research consistently emphasised both the importance and ongoing challenges of continuity of care during the transition from prisons to the community (Kouyoumdjian, Wiwcharuk and Green 2015; Carswell *et al*., 2017). For instance, an Australian medical record study showed that for female prisoners the transfer of health information from prison to community care was better in situations where a formal programme of information exchange existed, suggesting that outside such formal programmes transfer of health information is limited (Abbott *et al*., 2017a). While this study was based on the review of paper medical records, the use of electronic medical records – when containing good quality information –, may contribute to information exchange between prison health care and primary care. Poor integration between community and prison healthcare services will contribute to poor continuity of care, and such fragmentation of health care will subsequently affect both prisoners’ and public health outcomes.

The benefits of a well-organised primary care system in prison may extend beyond potential health benefits of (former) detainees. Reducing health inequalities by addressing the health needs and improving the health literacy of incarcerated individuals may also have an impact on more general rehabilitation goals such as preparing an individual for release. A good health and the ability to maintain it may enable an individual to be a more productive member of society on release (e.g. finding a job, being a good parent). This requires a change of focus of primary care in prison settings from acute health problems to the upstream causes of ill health and to involvement of patients in managing their own health (De Viggiani, 2006). Although primary care has a particularly important role here, given the barriers to accessing healthcare in the community, there is (to our knowledge) no research that specifically addresses this.

The Covid-19 pandemic has put particular strains on prison health care and the situation of prisoners. Effects of the pandemic have been felt heavy in the prison situation due to overcrowding, cramped living conditions, and lack of access to good quality care, both inside and outside the facility. Opportunities to work, relax, and exercise have been severely restricted (Hutchings and Davies, 2021).

In sum, while it is well-known that people who experience incarceration have high levels of mental and physical health problems, less is known about the organisation of, access to, and effects of primary health care for people who experience incarceration. Knowledge on facilitators and barriers, and best practices of organising and providing primary care in and after prison is of paramount importance because access to primary care has been linked to positive health outcomes both in general and incarcerated populations. Importantly, primary care providers have a significant opportunity to contribute to better continuity of care of people transitioning in and out of prisons.

## Experiences with prison care: Three cases

To illustrate some of the challenges in care for incarcerated individuals that have been mentioned above, we shortly discuss three case descriptions from three European countries. These cases have been presented during European Forum for Primary Care (EFPC) webinars and working group meetings as well. The first experience comes from the Kyrgyz Republic and illustrates the governance and organisational problems of prison health care. The second case describes the experiences of a Dutch (ex-) prisoner with problematic access to health care during incarceration and illustrates difficulties with triage and health care during out-of-office hours. The third case describes the difficulties of an ex-prisoner shortly after release and illustrates challenges related to continuity of care between the prison system and the health care in the community.

### The organisation of prison health care in the Kyrgyz Republic

Three Ministries and Services (the Ministries of Internal Affairs, of Justice, and of Health, and until recently the State Penal Service which is now an agency within the Ministry of Justice) are involved in healthcare provision to incarcerated individuals. The smooth work of these agencies requires substantial coordination, which is not always in place. Pre-trial detention centres and prisons are part of the State Penal Service and have their own medical departments. There are several difficulties related to the governance of the prison system. The first difficulty is the dual loyalty conflict; the clinical role conflict between professional duties to a patient and obligations to the prison administration. For example, prison administrations may pressure health care professionals to withhold evidence-based treatment that is available in the community due to financial or security reasons. Second, trust between prisoners and prison medical staff is endangered by internal prison rules that do not encourage interaction with the prison administration. Hence, prison doctors, who are part of the prison administration, face barriers when building doctor–patient relationships with those who need medical support. Another difficulty is related to the fact that the prison medical service works separate from public health care and has no direct access to the pre-detention health records of the patients. This information has to be requested, which requires both paperwork and time. Prison doctors are not controlled by the health authorities and do not have access to post-graduate education, nor to the latest clinical protocols. However, medical staff in prisons are eligible to earn military ranks, which implies higher salaries and early retirement. Undoubtedly, this makes prison work attractive for many doctors. Nevertheless, an assessment of prison healthcare made several years ago showed that primary healthcare and hospital treatment in prisons are of a lower standard than in the general community. In addition, many prison medical facilities lack qualified doctors. Even large correctional facilities with more than 1000 prisoners sometimes do not have any doctors but have to rely on nurses and paramedics only.

Within the Kyrgyz penal system there are two hospitals, one treating only TB patients and one general healthcare facility. Funding for both hospitals is provided by the Penal Authorities who have to operate under severe budget deficits. Most of the available money goes to custodial, security, salaries, and other priority items. As a consequence, the prison healthcare lacks medications and equipment, which contributes to a deplorable state of the medical departments.

The last stop on the way to release is an open-type settlement facility, where prisoners are allowed to go out of the facilities to encourage their reintegration. These open facilities have no medical departments; people are supposed to contact public healthcare institutions for care and treatment. However, in order to contact such an institution, ID documents are required which many marginalised people, including ex-prisoners, do not have. In addition, some prisoners are not allowed to go out of the facility due to violations of the prison rules or lack of a job. In this case, they need to inform the head of the facility that they need, for example, to go to the AIDS Centre. Most individuals are reluctant to do so because of stigma and discrimination. Moreover, in some cases, the facilities are located in remote areas limiting prisoners to access AIDS Centres and other specialised care institutions.

Finally, the state does not support any pre-release programs for prisoners that focus on continuity of treatment and care. Temporary housing, social support, and community reintegration services are provided mostly by local non-governmental organisations (NGOs). NGOs are very important in prison healthcare in the Kyrgyz Republic and the penal authorities are very open to cooperation with the non-governmental sector. In healthcare, such cooperation mostly takes place in the areas of drug dependence, HIV and TB. NGOs also work to bridge the gaps between public healthcare, prison healthcare, and the community.

### The case of Gerard, a prisoner in the Netherlands

In Dutch prisons, Psycho-Medical-Consultation teams – consisting of the institution’s psychologist, the psychiatrist, the doctor, and nurse – coordinate the basic health care for prisoners (i.e. screening, diagnostics, medication, and short-term structuring treatment). If basic care is insufficient, prisoners can be referred to more specialised medical or mental treatment in either the Judicial Centre for Somatic Care, general hospitals in the community, general forensic clinics or to one of the penitentiary psychiatric centres (for those with severe mental health problems). In general, detainees with a health need contact the prison nurse who is responsible for triage and decides on the need for referrals. In Dutch prisons, health care providers are only available during office hours. When an emergency happens after office hours, the prison officer is in charge of triage; he/she decides to call a doctor of the regional public health service or to call 112.

This case tells the story of Gerard – not a pseudonym; he wanted to use his real name – as he presented it during one of the EFPC webinars on prison health care. Towards an evening, Gerard slipped in his cell and felt a pain in his foot. With no after-hours medical care available, he filled out a note requesting a consultation the next day. The nurse, who is responsible for triage made an initial assessment, observed a sprain and did not refer Gerard to a doctor. After 3 days, in which he experienced a lot of pain and made repeated requests for further examination, Gerard was seen by a doctor. The doctor came up with the same diagnosis, a sprain, and according to the doctor it was not necessary to do an X-ray. An X-ray requires quite a lot of organisation and a special, secured transport to a hospital.

Meanwhile Gerard’s foot was not doing well; it became swollen, it became inflamed, and further investigation had to take place. Finally, after more than 2 weeks an X-ray showed a complicated fracture that needed surgery. Due to the delayed treatment, surgery, and quite a few hospitalisations, Gerard still experiences pain and infections in his foot after 1 and a half years, and there is a doomsday scenario hanging over his head of amputation.

### The case of Ed, a prisoner in England

The following is an outline of the case of a male who was released from a category B prison in England. The case has been provided by a service offering post-release support. Whilst this is a single case, the situation is not an isolated instance.

Ed – a pseudonym was used – had a diagnosis of schizoaffective disorder, for which he received daily medication. He had been released from custody part way through his sentence on a probation licence. At the time of release, he had spent 17 months in custody and had been stable on his medication and engaging well with services. Ed usually collected his medication from the medications hatch on his residential wing. On the day of his release, Ed was picked up from his cell very early and led straight to the reception. He did not realise he needed to collect his medication for post-release from the wing as he would have done on a normal day, and no one had informed him to do so. During his pre-release interviews, which included a brief chat with healthcare, no one spoke to him about the need to collect his medication. Moreover, during his pre-release checks at the prison reception, nobody checked if he needed and had been provided with post-release medication.

Ed was met by a mentor immediately after his release. The mentor was from a non-statutory agency and had been asked by the prison chaplaincy team to support Ed on his day of release to attend appointments with probation and housing. During his first appointment, Ed realised that he did not have his medication and became very stressed. The mentor contacted the prison on Ed’s behalf and they said they could not help because he had already left the facility. Ed contacted his GP himself, by phone, and explained the situation. They told him they had no appointments available for 1 week. Ed became increasingly exasperated by the situation. His mentor then phoned the GP to advocate on Ed’s behalf. She managed to get an appointment the same day and Ed was provided with medication. Had Ed not have had a mentor with him, the outcome could have been very different. However, the situation could have been avoided if someone had informed Ed about the need to pick up his post-release medications, had directed Ed to the medications hatch, or had checked whether or not he had his required medication.

## The position of EFPC

This position paper was drafted by a core group on behalf of the EFPC working group on prison health. It was discussed on several webinars and with the members of the EFPC. Finally, the position taken was endorsed by the Board of EFPC. In line with the three pillars of EFPC – professionals and their practice, policy, and research – the recommendations also address these three areas. Before doing so, we address some general issues.

### Basic principles for good prison health

#### Involvement of (former) prisoners

Starting point is that we should involve (former) detainees and their family, not only in care provision, but also especially in developing policies and designing research on care in prisons. Instruments to involve prisoners and let them ‘co-create’ the prison health care can be developed in cooperation with NGOs to support prisoners to provide insights and feedback. In health care research, it is already increasingly common to involve patients (Scheffelaar *et al*., 2020).

#### Equivalence and equity

As mentioned above, the basic normative principle is that of equivalence of care in prisons: care in prisons should be the same as in the community. However, it should be added that another important principle is that of equity: equal care for equal need. Combining these two principles, it may be necessary to provide more and or different kinds of primary care to (ex-) prisoner populations, taking their elevated health needs into account.

#### Opportunities to improve health

A good prison healthcare system provides an important opportunity to address poor health, health behaviours, self-management capacities, and health literacy and reduce health inequalities. The prison healthcare system has the potential to contribute to the individual health of prisoners and to public health by identifying health needs in prison; providing high quality care in prison; and integrating prison health care into the continuum of care in community health care services (McLeod *et al*., 2020).

#### Basic conditions of confinement

The conditions in detention should as much as possible contribute to health and should not compromise health. Such conditions include enough space and no overcrowding, enough light and fresh air, good hygiene and a clean environment, and adequate nutrition (UN Office on Drugs and Crime, 2013).

### Primary care professionals: what should be changed in day to day primary care practice during and after incarceration?

#### Awareness

Primary care providers in the community should be aware that part of their patient population consists of former prisoners who may have special health needs and may have experiences that influence their attitudes towards health and health care. However, at the same time, former prisoners may not want to disclose recent detention spells to primary care providers or even refrain from visiting them due to shame and fear of stigma or differential treatment (Abbott *et al*., 2017b). Primary care providers inside prisons should be aware of the potential tension between the requirements of good patient care and the demands from the prison organisation/management.

#### Respectful and open-minded interaction

It is important that primary care workers, both in the community and in prisons, try to build a relationship of trust with people who experience incarceration and their families, and have a non-judgmental and non-stigmatising approach (Kinner *et al*., 2015). It should be realised that the disempowerment of people during detention may be detrimental to self-management and shared decision-making (Kinner *et al*., 2015). The relationship between detainees and their care providers should be safe at all times.

#### Person-centred care

Person-centredness should be central in prison care. This may be hampered by processes at the patient side and at the prison organisation side. At the patient side, secondary illness benefits perhaps play a stronger role inside than outside prisons. At the side of the prison organisation, there may be pressures to organise health care as a rational management process. Both may be detrimental to the attention for person-centred care.

#### Professional competencies

Prison care professionals need the appropriate (post-graduate) education to be able to work in the prison context and to acquire adequate knowledge to treat common problems in the prison population, such as substance use, mental health problems, and communicable diseases. When only nurses are available, they should be trained in triage as well. Adequate training is a shared responsibility of the professionals working in prisons and the management and policies surrounding prison care.

#### Treatment and prevention

Given the health problems of incarcerated individuals and the link of many of their health problems to their life style, there should be equal attention to treatment and prevention, including health promotion. Often the focus is only on acute care or on urgent care that cannot be delayed. The use of eHealth solutions should be further explored (Tian *et al*., 2021).

#### Continuity of care

Continuity of care is of utmost importance. Incarceration and the transitions between prisons and the community provide a unique opportunity to improve continuity of care by getting into contact with a usually hard to reach vulnerable group, by identifying and addressing their health needs during detention, and by facilitating access to health care for this vulnerable group both in prison and after release by referring them to relevant healthcare services. In this regard, primary care providers in the community and in penitentiary facilities may provide an important opportunity to establish and strengthen ties with community health services and social services, which will contribute to the health and well-being of people who are released from prison. Continuity of care includes the links to social care. NGOs support prisoners during release to ensure continuation of treatment and care and primary care providers should have a social map of organisations and institutes to refer to.

### Policy makers: What should be changed in primary care policy?

#### Policy agenda

Just as among health care professionals, awareness among policy-makers (and politicians) about the specific health needs of prison populations is important. The current political culture tends to depict incarceration as an individual problem. However, the social characteristics of prison populations across countries show a social patterning, which demands for a more societal perspective and approach. Prevention of reoffending and reincarceration is an important aim of imprisonment, and adequate public health and health care and continuity of care could contribute to such prevention of recidivism (MacDonald *et al*., 2012).

#### Governance

Prison health care is generally governed by ministries of health and ministries of justice. It is recommended that the ministry of health is responsible for prison health care because this ensures that health care in prisons is as much as possible independent of the interests of the prison organisation, and avoids role conflict among health care professionals.

#### Organisation of care

The equivalence principles and the focus on health needs should guide the organisation of prison primary care. An adequate team skill mix should be available: prison primary care should include specialised primary medical care and nursing care, life style and preventive care, and should have close links to social care as well.

#### Accessibility

There should be adequate access to primary care in prisons. Capacity should be organised in such a way that adequate care can also be provided during out-of-office hours. Referrals to specialised care and hospitals should be possible and the prison organisation should facilitate such transport.

#### Integrated care

A good skill mix of prison health and social care teams facilitates integrated care. Mental health care and addiction care should be integrated in prison primary care. Moreover, post-prison primary care and social care should be well-connected to the prison care system.

#### Transfer policy

Policies should be designed to facilitate the smooth transfer from prison health care to primary care and community care. From the policies for smooth transfer from hospital care to primary and community care, it may be concluded that a dedicated function of transfer nurse is an option. Transfer policies should include the transfer of medical information between prisons and to community primary care and specialist care, if relevant and approved by the (ex-)prisoner in question.

### Researchers and research funders: what can research contribute?

#### Dedicated research programmes

To the best of our knowledge, research on (primary) health care in prisons (in contrast to health of prisoners) is a largely neglected area. Not only politically, but also from the side of research programmers, funders, and researchers not much attention has been paid to prison health care. The research that does exist seems fragmented and targeted either at the more general governance of care in prison or at specific health care interventions. As a consequence, knowledge on care in prisons, and in particular primary care – its organisation, quality, and effects – is limited. Still, the wide range of organisational arrangements of prison health care provides opportunities to learn from each other.

#### Focus on organisation of care

Research should not only focus on the effectiveness of specific interventions (however important these are), but also – and mainly – on the organisation and quality of (primary) care in prisons, in relation to the organisation and quality of care in the outside world. Important questions are how continuity of care and information exchange between prison and community can be improved. This also requires the development of indicators for quality of care.

#### Data availability

Data from (electronic) medical records should be made available for quality indicator development, monitoring health care, and for research purposes in a privacy-responsible and protected environment, in accordance with the General Data Protection regulation and other legislation.

#### Importance of context

Comparative research on the organisation of care should take the context of the national health care systems into account for two reasons. First, the organisation of prison health inherently linked to that of the wider health care system. And secondly, to be able to learn from research performed in other countries, insight in the different contexts is important.

#### International comparative research

To learn from different experiences, a research programme on prison health care should compare the situation in different (European) countries. This requires a dedicated investment from a supra-national body, such as the EU in their upcoming framework programme Horizon Europe.

## Conclusions

Prisoners and ex-prisoners have a worse physical and mental health compared with a cross-section of the population. However, access to good quality treatment and care is often worse than in the outside situation. In particular, well-organised primary care in the prison context could benefit prisoners and, indirectly, society at large. Moreover, continuity of care between the community and the prison situation needs improvement.

## Footnotes

1 This paper focuses on all persons who are placed in a correctional facility and experience a detention period (i.e. both detainees in pre-trial detention and those sentenced to a prison sentence).
